# Sleepless in inequality: findings from the 2018 behavioral risk factor surveillance system, a cross-sectional study

**DOI:** 10.1186/s12889-022-14292-5

**Published:** 2022-10-27

**Authors:** Roman Pabayo, Priya Patel, Sze Y. Liu, Beth E. Molnar

**Affiliations:** 1 University of Alberta School of Public Health, T6G 2R3 3-300 Edmonton Clinic Health Academy, 11405-87 Ave., Edmonton, Alberta Canada; 2 Public Health Department, Montclair State University, New Jersey, USA; 3 Bouvé College of Health Sciences, Northeastern University, Boston, MA USA

**Keywords:** Sleep, Income inequality, Social and health inequities

## Abstract

**Background:**

Despite the large body of research on the adverse effects of income inequality, to date, few studies have examined its impact on sleep. The objective of this investigation is to examine the association between US state income inequality and the odds for regularly obtaining inadequate (< 7 h) and very inadequate (< 5 h) of sleep in the last 24 h.

**Methods:**

We analysed data from 350,929 adults participating in the US 2018 Behavioral Risk Factor Surveillance System (BRFSS). Multilevel modeling was used to determine the association between state-level income inequality, as measured by the Gini coefficient, and the odds for obtaining inadequate and very inadequate sleep. We also determined if associations were heterogeneous across gender.

**Results:**

A standard deviation increase in the Gini coefficient was associated with increased odds for inadequate (OR = 1.06, 95% CI: 1.00, 1.13) and very inadequate sleep (OR = 1.11, 95% CI: 1.03,1.20). Also, a cross-level Gini Coefficient X Gender interaction term was significant (OR = 1.07, 95% CI:1.01,1.13), indicating that increasing income inequality was more detrimental to women’s sleep behavior.

**Conclusion:**

Future work should be conducted to determine whether decreasing the wide gap between incomes can alleviate the burden of income inequality on inadequate sleep in the United States.

## Background

Approximately a third of adults in the US do not regularly achieve the recommended 7 to 9 h of sleep [1]. Moreover, sleep deprivation in the US is becoming increasingly prevalent. Americans aged 18 to 84 showed a 15% increase in the prevalence of short sleep (less than or equal to 6 h) from 2013 to 2017 [2]. Short-term sleep deprivation has been associated with increased fatigue and irritability, and decreased working memory, attention, and processing speed [3]. Prolonged or chronic sleep deprivation, in addition to amplifying the consequences of short term sleep deprivation, is associated with an increased risk for mental illness including depression and anxiety [4, 5], increased risk of physical aggression against peers among urban youth [6], and increased risk for chronic disease such as hypertension, diabetes, and cancer [7–11], all of which contribute to increased mortality and exert a significant financial toll on America’s healthcare system [7, 12]. Also, sleep deprivation in the US is estimated to cost $411 billion annually in lost worker productivity [13].

Evidence suggests that certain groups are disproportionately less likely to obtain sufficient sleep for optimal growth and health. African-Americans, Hispanic and Latino Americans, people who smoke, are sedentary and obese, and those with low household incomes [14, 15] are less likely to obtain the recommended hours of sleep. Women and older adults are more susceptible to sleep disorders that may contribute to sleep deprivation [16] [17]. However, in the nationally representative Behavioral Risk Factor Surveillance System (BRFSS) of adults in the U.S., no gender differences in short sleep duration are typically observed and older adults have the lowest prevalence of sleep deprivation relative to younger age groups [14].

In addition to individual-level risk factors, several physical neighborhood risk factors for sleep deprivation have been identified. For example, a study of 300,000 Americans aged 51 to 71 years found that the risk of sleeping less than 5 h increased by 46% and 72% in men and women, respectively, who lived in neighborhoods in the lowest socioeconomic status quintile, relative to those who lived in neighbourhoods in the highest quintile [18]. Sleep deprivation among urban youth in Boston, Massachusetts, U.S.A. was associated with higher neighborhood concentrated poverty [19]. Neighbourhood noise in the form of traffic noise has also been linked to sleep disruption [20] [21]. Moreover, social fragmentation, the lack of connectedness between individuals and society, has been shown to increase the risk of sleep deprivation in American youth [22].

Beyond the physical environment, a neighborhood’s social environment may also affect sleep deprivation. Income inequality, the disparity between rich and poor within a society, is an understudied potential risk factor of sleep deprivation. One possible mechanism in which income inequality may influence sleep is the psychosocial theory wherein increasing income inequality may exacerbate feelings of insecurity among community members who feel that they have been “left behind”[23]. This may contribute to the documented link between income inequality and increased risk of depression [24], a common risk factor for sleep deprivation [25]. Second, evidence indicates income inequality is associated with a decrease in social cohesion, which is the feelings of connectedness and solidarity among members living within a community [26]. Social cohesion is shown to be protective against mental health conditions such as depression and anxiety [22] [27], which in turn, can affect sleep [25]. To our knowledge, only one study has examined the role of income inequality in sleep behaviour. Clement et al., using data from the Mexican Health and Nutrition Survey, found an inverse correlation between municipal-level income inequality and quality of sleep [28].

Previous research has indicated that income inequality may be differentially associated with mental health outcomes between men and women [28–30]. For example, income inequality was associated with risk for major depression among US women [30] and for higher depressive symptoms among adolescent girls [29]. Also, income inequality at the municipal-level was associated with quality of sleep among women but not men [28]. One possible explanation for heterogeneity across genders is that women are more influenced by the erosion of social cohesion resulting from income inequality [31].

An improved understanding of the association between income inequality and inadequate sleep duration may provide policy-relevant insight into developing interventions to promote healthier levels of sleep. Therefore, using data from the Behavioral Risk Factor Surveillance System (BRFSS) [14], the current study aimed to assess the association between neighbourhood-level income inequality and adult sleep duration. Based on prior literature, we hypothesize increasing income inequality is related to an increased odds of obtaining inadequate sleep. Furthermore, we test whether depression is a potential mediator in this relationship.

## Methods

Data for this investigation came from the 2018 Behavioral Risk Factor Surveillance System (BRFSS), a random-digit dialed telephone survey conducted annually by the Centers for Disease Control and Prevention. Of the 437,436 respondents, 62.2% (n = 272,154) completed the questionnaire via cellular phone [32]. The BRFSS collects health behavior and risk data from all 50 states and the District of Columbia and has been described elsewhere [32]. The study population includes non-institutionalized individuals aged 18 and older with access to a landline or a cellular telephone. Design weights were developed to take into account the BRFSS survey’s design and the population’s characteristics. When applied, data weighting helps make sample data more representative of the U.S. adult population from which the data were collected [32]. The data utilized for this study are available in https://www.cdc.gov/brfss/annual_data/annual_2018.html.

### Measures

#### Area-level covariates

The exposure of interest is income inequality, or the degree of income disparity, within each of the 50 U.S. states and the District of Columbia, which was measured using the Gini coefficient. The Gini coefficient ranges from 0 (perfect equality, indicating little gaps between rich and poor and every household earns the same income) to 1.0 (perfect inequality, indicating large gaps between rich and poor) [33]. Other state-level covariates include median income, proportion living in poverty, proportion that is Black, and population size. Continuous measures of state-level covariates were standardized using the Z-transformation.

### Individual-level covariates

Individual-level covariates that may confound the relationship between income inequality and sleep behavior include gender, age, race, and education (less than high school, high school, some college, and college graduate), and marital status (coupled or single). Using tertile thresholds, total household income was categorized into low (less than $35,000), medium ($35,000 to $75,000), and high (greater than $75,000).

### Outcome measures

We created two sleep behavior outcome variables based on participants’ responses to the question measuring the number of hours of sleep obtained in a 24-hour period, on average [32]. Those who reported sleeping less than 7 h were categorized as getting *inadequate sleep* [32]. We also tested a threshold of less than 5 h of sleep. Those who reported less than 5 h were categorized as receiving *very inadequate sleep.* Respondents who did not know, were unsure, refused, or had a missing response were excluded. The test-rest reliability was 0.89, while criterion and convergent/discriminant validity were deemed acceptable [34].

### Statistical analyses

Because BRFSS participants were nested within 50 states and the District of Columbia, multilevel logistic regression modeling was used to investigate the potential relation between state-level income inequality, as measured by the Gini coefficient, and having inadequate and very inadequate sleep. A sequence of pre-specified models was conducted. First, we estimated a state-level intercept-only model, which allowed us to calculate the overall predicted probability and the plausible value range. The plausible value range, similar to the ICC, allows us to calculate the degree of variability of inadequate and very inadequate sleep across the states. For example, the range presents the minimum and maximum values in proportions of respondents obtaining inadequate and very inadequate sleep. Second, we estimated the unadjusted association between the Gini coefficient and the odds for obtaining inadequate and very inadequate sleep. Third, we added state-level and individual-level covariates in the models. Fourth, we tested cross-level interaction terms, Gender x Gini coefficient, to test if associations between income inequality and the odds for obtaining inadequate and very inadequate sleep were heterogeneous across gender. Gini coefficient and race and household income cross-level interaction terms were also tested but not significant (results not presented). The 2018 Behavioral Risk Factor Surveillance System sampling weights were used to reduce potential selection bias, and thus make estimates more generalizable to the population. Analyses were conducted using Stata v. 14.0.

To determine whether experiencing poor mental health days acted as a mediator between state-level income inequality and sleep, we adjusted for the number of days each respondent’s mental health was not good. In the presence of mediation, the association between income inequality and inadequate and very inadequate sleep would be expected to be attenuated. Then, we applied the Baron and Kenny method to test for mediation [35]. We assessed the following bivariate associations: (1) state-level Gini coefficient and the number of days the respondent’s mental health was not good; (2) state-level income inequality and each of the sleep outcomes (< 7 h and < 5 h) controlling for the possible mediating variable; (3) the number of days the respondent’s mental health was not good and the two sleep outcomes.

## Results

The 2018 Behavioral Risk Factor Surveillance System dataset included 425,712 respondents from 50 states and the District of Columbia. All respondents with missing data on sleep behavior and other covariates were excluded, resulting in a case-complete dataset of 350,929 individuals (82.4%). Participants removed were more likely to be Black Non-Hispanic, male, younger in age, and from rural settings.

Table 1 presents the characteristics and the corresponding weighted percentage of the respondents with complete data. Among the respondents, 50.0% were women. A majority of the participants were white (64.6%), followed by Hispanic (15.8%), and Black (11.6%). Of the sample, 36.1%, 28.2%, and 35.7% were from high, medium, and low household income backgrounds, respectively. Most of the respondents lived in an urban setting (93.5%).

**Table 1:** Characteristics of US adults participating in the 2018 Behavioral Risk Factor Surveillance System (BRFSS) (n = 350,929) and US states (50 states and the District of Columbia)

Individual Level Characteristics	Unweighted n	Weighted %	
Gender			
Male	164,440	50.0	
Female	186,389	50.0	
Age, years			
18–24	18,548	11.0	
25–44	84,215	35.5	
45–64	131,220	34.0	
65 and older	116,946	19.5	
Racial Background			
White, Non-Hispanic	271,245	64.6	
Black, Non-Hispanic	28,809	11.6	
Hispanic	25,730	15.8	
Asian, Non-Hispanic	7,815	5.1	
American Indian/Alaskan Native, Non-Hispanic	6,758	1.1	
Other race, Non-Hispanic	10,752	2.0	
Household Income			
Low	123,527	35.7	
Medium	105,766	28.2	
High	121,636	36.1	
Education			
Less than High School	22,974	11.9	
High School	91,852	27.0	
Some College	97,605	31.6	
College	138,498	29.6	
Marital Status			
Couple	197,728	56.8	
Single	153,201	43.2	
Setting			
Urban	297,690	93.5	
Rural	53,239	6.5	
State Level Characteristics (n = 51)	Mean (SD)	Median	Range
Gini Coefficient	0.468(0.02)	0.468	0.427–0.524
State Median Income, USD	58,143(9,820)	56,565	41,754 − 78,9945
Proportion Black	10.9	6.9	0.6–46.8
Proportion Poor	22.5(13.1)	23.0	1.0–45.0
State Population	6,332,183 (7,235,904)	4,438,182	584,215 − 39,167,117

The characteristics of the 50 states and Districts of Columbia are also described in Table 1. The Gini Index had a mean of 0.468, a standard deviation of 0.02, a median of 0.468, and ranged from 0.427 to 0.524. The State median income was $58,143 (SD = 9,820), the mean proportion Black was 10.9% (SD = 10.7), the mean proportion poor was 22.5% (SD = 13.1), and the mean population was 6,332,183 (SD = 7,235,904).

The intercept-only model indicated that the overall predicted probability was 36.2% and 4.6% for inadequate and very inadequate sleep, respectively. Also, the intercept-only model confirmed significant variability in the percentage of the population obtaining less than 7 h and less than 5 h of sleep regularly every day. For example, the overall predictive probability was 30.7–42.2% and 3.2–5.5% for inadequate and very inadequate sleep across US states.

The crude bivariate and adjusted associations are presented in Table 2. In the adjusted analyses, in comparison to men, women were less likely to obtain inadequate (OR = 0.94, 95% CI: 0.92,0.96) and very inadequate sleep (OR = 0.91, 95% CI:0.84,0.98) (Table 2). Also, those from low household incomes were more likely to obtain inadequate (OR = 1.14, 95%CI: 1.07, 1.21) and very inadequate sleep (OR = 2.10, 95% CI:1.88, 2.34), in comparison to those from high household incomes.

**Table 2:** Cross-sectional associations between income inequality and odds for obtaining inadequate (< 7 h of sleep) and very inadequate (< 5 h of sleep), among participants in the 2018 Behavioral Risk Factor Surveillance System (BRFSS)

	< 7 h of sleep						< 5 h of sleep							
	Crude Bivariate		Adjusted*		Adjusted + Interaction**			Crude Bivariate		Adjusted*		Adjusted + Interaction**		
	**OR**	**95%CI**	**OR**	**95%CI**	**OR**	**95%CI**		**OR**	**95%CI**	**OR**	**95%CI**	**OR**	**95%CI**	
Intercept	0.56	(0.54, 0.58)	0.43	(0.36, 0.50)	0.43	(0.36, 0.50)		0.05	(0.04, 0.05)	0.03	(0.03, 0.04)	0.03	(0.03, 0.04)	
**State Characteristics**														
Gini (Z-Score)	1.06	(1.01, 1.11)	1.06	(1.00, 1.13)	1.05	(0.99, 1.12)		1.08	(0.99, 1.17)	1.11	(1.03, 1.20)	1.08	(0.99, 1.16)	
State Median Income (Z-Score)	0.96	(0.91,1.01)	0.98	(0.93, 1.03)	0.98	(0.93, 1.03)		0.90	(0.84,0.97)	0.96	(0.88, 1.05)	0.96	(0.88, 1.05)	
Population Size (Z-Score)	1.00	(0.98,1.01)	0.99	(0.96, 1.02)	0.99	(0.96, 1.02)		0.97	(0.93,1.01)	0.96	(0.92, 1.01)	0.96	(0.92, 1.01)	
Proportion Black (Z-Score)	1.08	(1.05,1.11)	1.00	(0.96, 1.05)	1.00	(0.96, 1.05)		1.13	(1.07,1.20)	1.01	(0.95, 1.08)	1.01	(0.95, 1.08)	
Proportion in Poverty (Z-Score)	1.05	(0.98,1.12)	0.99	(0.93, 1.05)	0.99	(0.93, 1.05)		1.09	(1.01,1.19)	0.96	(0.86, 1.08)	0.96	(0.86, 1.08)	
**Individual Characteristics**														
Gender (ref: male)														
Female	0.95	(0.93,0.97)	0.94	(0.92, 0.96)	0.93	(0.91, 0.95)		0.95	(0.89,1.03)	0.91	(0.84, 0.98)	0.88	(0.82, 0.94)	
Gini Z-Score					1.01	(0.99, 1.03)						1.07	(1.01, 1.13)	
Age (ref: 18 to 24 years)														
25 to 44 years	1.19	(1.13,1.26)	1.40	(1.31, 1.49)	1.40	(1.31, 1.49)		1.16	(1.05,1.27)	0.50	(1.35, 1.68)	1.50	(1.35, 1.68)	
45 to 64 years	1.11	(1.06,1.16)	1.31	(1.22, 1.41)	1.31	(1.22,1.49)		1.20	(1.09,1.32)	1.58	(1.41, 1.78)	1.58	(1.41, 1.78)	
65 years and older	0.68	(0.63,0.75)	0.78	(0.69, 0.87)	0.78	(0.69, 0.87)		0.79	(0.68,0.93)	0.91	(0.75, 1.10)	0.91	(0.75, 1.10)	
Household Income (ref:: high)														
Medium	1.15	(1.11,1.20)	1.08	(1.04, 1.13)	1.08	(1.04, 1.13)		1.53	(1.40,1.68)	1.33	(1.21, 1.46)	1.33	(1.21, 1.46)	
Low	1.29	(1.20,1.38)	1.14	(1.07, 1.21)	1.14	(1.07,1.21)		2.85	(2.56,3.18)	2.10	(1.88, 2.34)	2.10	(1.88, 2.34)	
Education (ref: no high school)														
High school	1.06	(0.96,1.16)	1.07	(0.99, 1.15)	1.07	(0.99, 1.15)		0.70	(0.50,0.73)	0.77	(0.65, 0.92)	0.77	(0.65, 0.92)	
Attended college	1.08	(0.96,1.21)	1.10	(1.00, 1.20)	1.10	(1.00, 1.20)		0.60	(0.50,0.73)	0.73	(0.62, 0.87)	0.73	(0.62, 0.87)	
College Graduate	0.75	(0.66,0.86)	0.78	(0.70, 0.86)	0.78	(0.70, 0.86)		0.31	(0.24,0.38)	0.45	(0.37, 0.54)	0.45	(0.37, 0.54)	
Race (ref: White, Non-Hispanic)														
Black, Non-Hispanic	1.71	(1.60,1.82)	1.49	(1.39, 1.61)	1.49	(1.39, 1.61)		1.77	(1.63,1.92)	1.28	(1.17, 1.40)	1.28	(1.17, 1.40)	
Asian, Non-Hispanic	1.27	(1.02,1.60)	1.32	(1.02, 1.69)	1.32	(1.02, 1.69)		1.17	(1.01,1.34)	1.31	(1.14, 1.52)	1.31	(1.14, 1.52)	
American Indian/Alaskan Native, Non-Hispanic	1.53	(1.38,1.70)	1.35	(1.20, 1.51)	1.35	(1.20, 1.51)		2.37	(1.93,2.91)	1.67	(1.32, 2.12)	1.67	(1.32, 2.12)	
Hispanic	1.08	(1.03,1.14)	0.92	(0.86, 0.98)	0.92	(0.86, 0.98)		1.24	(1.12,1.36)	0.80	(0.71, 0.90)	0.80	(0.71, 0.90)	
Other race, Non-Hispanic	1.60	(1.49,1.72)	1.47	(1.36, 1.58)	1.46	(1.36, 1.58)		2.17	(1.98,2.38)	1.85	(1.65, 2.07)	1.85	(1.65, 2.07)	
Marital status (ref: coupled)														
Single	1.26	(1.23,1.29)	1.18	(1.14, 1.22)	1.18	(1.14, 1.22)		1.74	(1.65,1.83)	1.38	(1.28, 1.48)	1.38	(1.28, 1.48)	
Setting (ref: Rural)														
Urban	0.96	(0.90,1.02)	0.96	(0.89, 1.03)	0.96	(0.89, 1.03)		0.77	(0.67,0.87)	0.85	(0.74, 0.98)	0.85	(0.74, 0.98)	

* Model includes State-level (Gini Z-score, Median-Income, Population Size, Proportion Black, Population in Poverty) Individual-level (Gender, Age, Household Income, Education, Race, Marital Status, Setting)

** Model includes State-level (Gini Z-score, Median-Income, Population Size, Proportion Black, Population in Poverty) Individual-level (Gender, Age, Household Income, Education, Race, Marital Status, Setting) and Gini Coefficient X Gender Cross-level Interaction

**Fig. 1:**
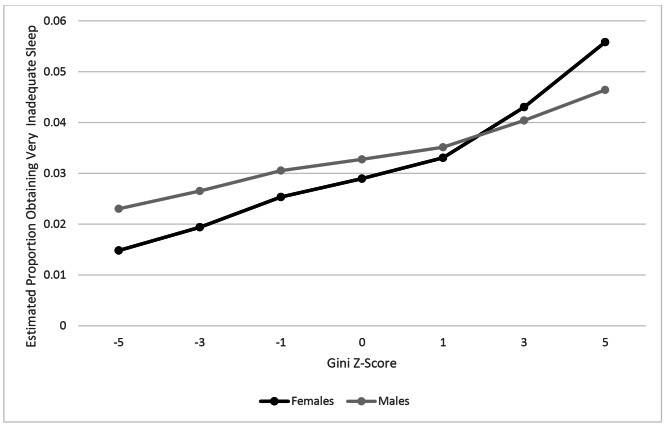
Association between Gini Index and obtaining very inadequate sleep (< 5 h/day)

Table 2 shows the association between the Gini coefficient and the odds of obtaining inadequate and very inadequate sleep. Crude analyses indicated that a standard deviation increase in Gini was associated with both an increased odds of obtaining inadequate sleep (OR = 1.06, 95% CI = 1.01, 1.11) and very inadequate sleep (OR = 1.08, 95% CI = 0.99, 1.17). Associations between income inequality and the odds for obtaining inadequate (OR = 1.06, 95% CI = 1.00, 1.13) and very inadequate (OR = 1.11, 95% CI = 1.03,1.20) hours of sleep remained after adjusting for individual and area level covariates. When testing to determine if the associations varied across genders (male vs. female), there was no heterogeneity when inadequate sleep was the outcome (1.01, 95% CI = 0.99,1.03). However, the cross-level interaction term indicated that a standard deviation unit increase in the Gini coefficient was associated with a further increased odds of receiving less than 5 h of sleep among women (OR = 1.07, 95% CI: 1.01,1.13). In other words, the estimated proportion of women obtaining less than 5 h of sleep is higher than the estimated proportion of men, particularly at higher levels of income inequality (Fig. 1).

**Fig. 2:**
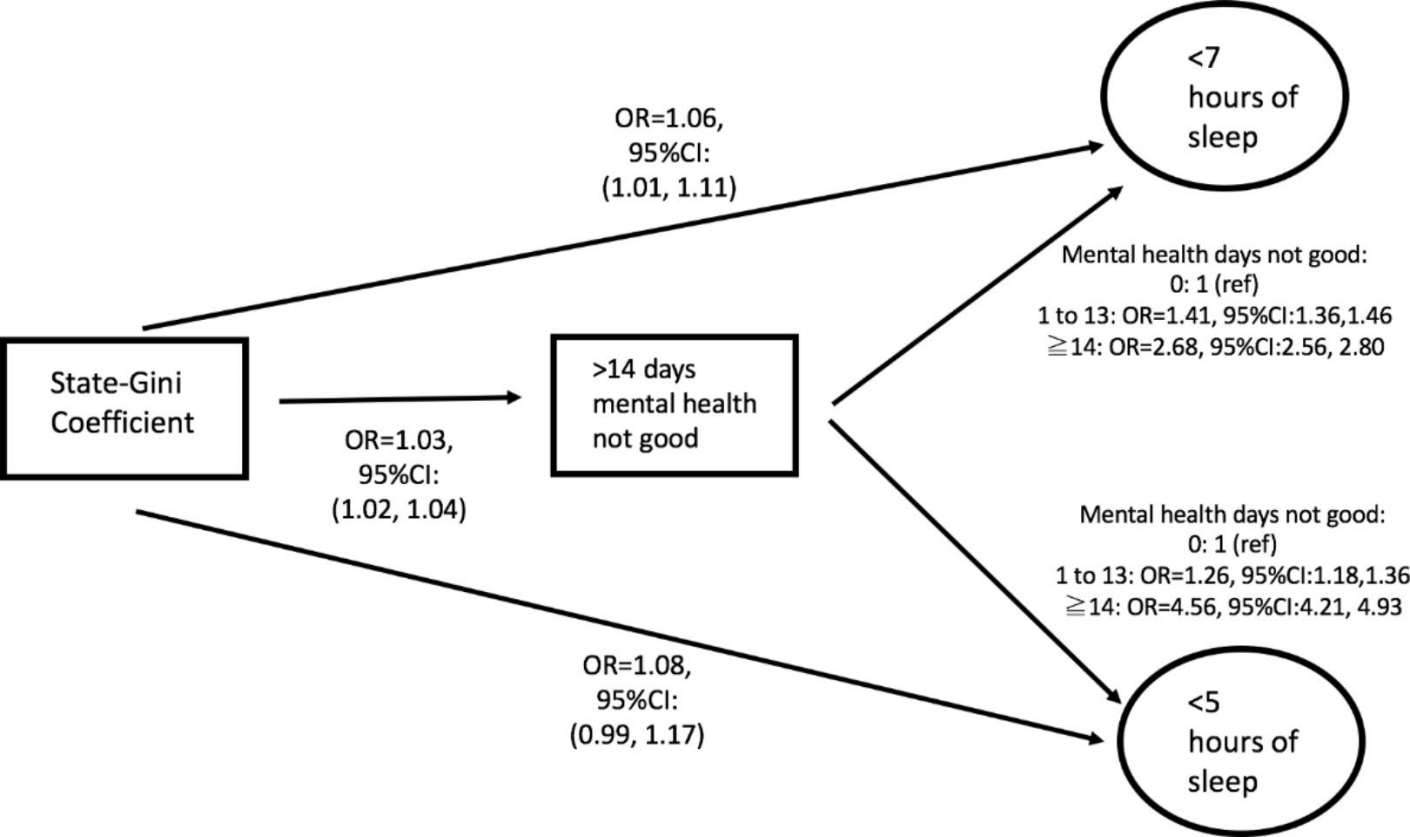
Associations between income inequality, mental health days not good (mediator), and < 7 h of sleep, and < 5 h of sleep

The addition of mediators (Table 3) resulted in a slight attenuation for the estimate for state-level Gini coefficient for inadequate sleep (OR = 1.06, 95% CI = 0.99, 1.12) and very inadequate sleep (OR = 1.09, 95% CI = 1.01, 1.17). Table 4 presents results from the Baron-Kenny mediation analyses examining bivariate associations. A standard deviation increase in state-level Gini coefficient was associated with an increased odds for obtaining inadequate sleep (OR = 1.06, 95% CI = 1.01, 1.11) and very inadequate sleep (OR = 1.08, 95% CI = 0.99, 1.17). Also, a standard deviation increase in state-level Gini coefficient is associated with an increase odds for experiencing 14 or more days of not good mental health in the previous month (OR = 1.03, 95% CI = 1.02, 1.04). Finally, in comparison to experiencing 0 days in which mental health was not good, those who had experienced 1 to 13 days and greater than and equal to 14 days in which mental health was not good, experienced greater odds for obtaining inadequate and very inadequate sleep. Figure 2 illustrates the observed mediating associations. Although direct and indirect pathways are depicted, these are proposed mechanisms and are not necessarily causal.

**Table 3:** Multilevel regression analyses while adjusting for mediator: number of days mental health not good among participants in the 2018 Behavioral Risk Factor Surveillance System (BRFSS)

	< 7 h of sleep				< 5 h of sleep			
	Adjusted		Adjusted + Interaction		Adjusted		Adjusted + Interaction	
	**OR**	**95%CI**	**OR**	**95%CI**	**OR**	**95%CI**	**OR**	**95%CI**
Intercept	0.34	(0.29, 0.41)	0.34	(0.29, 0.41)	0.02	(0.02, 0.03)	0.03	(0.02, 0.03)
**State Characteristics**								
Gini (Z-Score)	1.06	(0.99, 1.12)	1.04	(0.98, 1.11)	1.09	(1.01, 1.17)	1.05	(0.97, 1.12)
State Median Income (Z-Score)	0.98	(0.93, 1.03)	0.98	(0.93, 1.03)	0.97	(0.90, 1.05)	0.97	(0.90, 1.05)
Population Size (Z-Score)	0.99	(0.96, 1.02)	0.99	(0.96, 1.02)	0.97	(0.93, 1.02)	0.97	(0.93, 1.02)
Proportion Black (Z-Score)	1.01	(0.96, 1.05)	1.01	(0.96, 1.05)	1.02	(0.96, 1.09)	1.02	(0.96, 1.09)
Proportion in Poverty (Z-Score)	0.98	(0.92, 1.05)	0.98	(0.92, 1.05)	0.97	(0.87, 1.07)	0.97	(0.87, 1.07)
**Individual Characteristics**								
Gender (ref: male)								
Female	0.88	(0.86, 0.90)	0.87	(0.85, 0.89)	0.83	(0.77, 0.91)	0.80	(0.75, 0.86)
Gini Z-Score			1.02	(1.00, 1.04)			1.08	(1.03, 1.14)
Age (ref: 18 to 24 years)								
25 to 44 years	1.46	(1.36, 1.57)	1.46	(1.36, 1.57)	1.54	(1.37, 1.74)	1.55	(1.37, 1.74)
45 to 64 years	1.42	(1.30, 1.55)	1.42	(1.30, 1.55)	1.66	(1.46, 1.89)	1.66	(1.46, 1.89)
65 years and older	0.91	(0.80, 1.05)	0.91	(0.80, 1.05)	1.14	(0.94, 1.38)	1.14	(0.94, 1.38)
Household Income (ref:: high)								
Medium	1.04	(0.99, 1.10)	1.04	(0.99, 1.10)	1.23	(1.12, 1.36)	1.23	(1.11, 1.36)
Low	1.03	(0.97, 1.09)	1.03	(0.97, 1.09)	1.73	(1.56, 1.92)	1.73	(1.57, 1.92)
Education (ref: no high school)								
High school	1.11	(1.03, 1.19)	1.11	(1.03, 1.19)	0.83	(0.69, 0.99)	0.83	(0.69, 0.99)
Attended college	1.12	(1.02,1.22)	1.12	(1.02, 1.22)	0.77	(0.65, 0.91)	0.77	(0.65, 0.90)
College Graduate	0.80	(0.73, 0.89)	0.80	(0.73, 0.89)	0.49	(0.40, 0.59)	0.49	(0.40, 0.59)
Race (ref: White, Non-Hispanic)								
Black, Non-Hispanic	1.58	(1.47, 1.70)	1.58	(1.47, 1.70)	1.42	(1.31, 1.54)	1.42	(1.31, 1.54)
Asian, Non-Hispanic	1.42	(1.10, 1.82)	1.42	(1.10, 1.82)	1.54	(1.33, 1.78)	1.54	(1.33, 1.78)
American Indian/Alaskan Native, Non-Hispanic	1.37	(1.22, 1.54)	1.37	(1.22, 1.54)	1.72	(1.40, 2.13)	1.72	(1.39, 2.13)
Hispanic	1.00	(0.94, 1.05)	1.00	(0.94, 1.05)	0.94	(0.85, 1.04)	0.94	(0.85, 1.04)
Other race, Non-Hispanic	1.43	(1.33, 1.54)	1.43	(1.33, 1.54)	1.71	(1.55, 1.88)	1.71	(1.55, 1.88)
Marital status (ref: coupled)								
Single	1.13	(1.10, 1.17)	1.13	(1.10, 1.17)	1.27	(1.18, 1.37)	1.27	(1.18, 1.37)
Setting (ref: Rural)								
Urban	0.94	(0.87, 1.01)	0.94	(0.87, 1.01)	0.81	(0.70, 0.95)	0.81	(0.70, 0.95)
Number of days mental health not good (ref: none)								
1 to 13 days	1.40	(1.34, 1.46)	1.40	(1.34, 1.46)	1.29	(1.20, 1.38)	1.29	(1.20, 1.38)
≥ 14 days	2.54	(2.45, 2.63)	2.54	(2.45, 2.63)	3.84	(3.55, 4.16)	3.84	(3.55, 4.17)

## Discussion

### Main findings of this study

The objectives of this investigation were to determine if state-level income inequality was associated with the odds for obtaining inadequate sleep (less than 7 h) and very inadequate sleep (less than 5 h) per day. Also, we attempted to determine if any observed relationship was heterogeneous across gender. Finally, we determined if poor mental health acted as a mediator between state-level income inequality and sleep. Our multilevel analysis of population-based representative data collected in the US in 2018 suggests that state-level income inequality is associated with an increased likelihood of insufficient sleep. This relationship was also demonstrated to be more detrimental among women. Finally, experiencing poor mental health days may be a potential mediator between income inequality and inadequate sleep.

**Table 4:** Bivariate analysis of the relationships between potential mediators and Income Inequality and Inadequate and very Inadequate sleep

	Number of mental health days not good (> 14 days)	Inadequate Sleep (< 7 h)	Very Inadequate Sleep (< 5 h)
	OR (95% CI)	OR (95% CI)	
State-Gini	1.03(1.02, 1.04)	1.06(1.01, 1.11)	1.08(0.99,1.17)
*Possible Mediator*			
Number of days mental health not good (ref: none)		1.00	
1 to 13 days		1.41(1.36,1.46)	1.26(1.18,1.36)
≥ 14 days		2.68(2.56,2.80)	4.56(4.21,4.93)

### What is already known on this topic

Our results are consistent with previous work. For example, in a nationally representative household survey conducted in Mexico, municipal income inequality was significantly associated with lower sleep quality [28]. Similarly, in a large and nationally representative dataset conducted in Germany, a 10% increase in the income of relevant others, which is an individual-level measure of relative income inequality, is associated with a 6–8 min decrease in a person’s weekly amount of sleep, on average [36]. This investigation provides the first empirical evidence that income inequality impacts the amount of sleep among a representative sample of U.S. adults. Given that the US has greater higher income inequality than Mexico or Germany, the relationship may be more pronounced in the U.S.

### What this study adds

Although it has been proposed that adverse mental health outcomes, such as depression and anxiety may act as mediators between income inequality and insufficient sleep, another potential explanation is that inadequate sleep and other sleep problems, may be a marker for such mental health conditions. For example, the DSM-V criteria for depression includes sleep difficulties [37], described as “insomnia or hypersomnia nearly every day” as one of the possible symptoms, which is why a common indicator in depression measurements, such as the Center for Epidemiologic Studies Depression Scale (CESD-R), is having trouble getting to sleep. Furthermore, numerous studies have identified inadequate sleep as a risk factor for mental health conditions such as depression and anxiety [7, 38, 39]. Nonetheless, this investigation provides robust results that indicates income inequality is associated with inadequate sleep.

Findings indicate that the association between income inequality and odds for obtaining inadequate sleep is heterogeneous across men and women. Based on our results, adult women in the U.S. are less likely to obtain inadequate sleep, which corroborates other previous research [40]. However, increasing income inequality decreases this disparity since a higher Gini index is associated with an increased likelihood for inadequate sleep among women and not among men. This difference in association across genders is in agreement with previous work that indicates women are more likely to be detrimentally impacted by income inequality [22].

This study suggests that public health interventions to decrease income inequality may also alleviate the burden of inadequate sleep, especially among those living in high income inequality areas and those with living with depression and anxiety. By reducing the income gap between individuals in a society, the potential detrimental impacts of inequality can be abated, adding improvements in sleep to a list of other impacts including depression, anxiety, and aggression.

### Limitations of this study

Several study limitations have been identified. First, the study design utilized was cross-sectional, so temporality and causation cannot be inferred. Nonetheless, this investigation is one of the first to identify the relationship between income inequality and the odds for inadequate sleep among a nationally representative sample of adults in the U.S. Future research should include analysis of this relationship utilizing longitudinal data. Second, sleep behavior was measured via self-report, which can lead to a measurement bias.

## Conclusion

In summary, we observed a significant relationship between state-level income inequality with the odds of obtaining inadequate sleep, particularly among women. In states with high income inequality, an increase in standard deviation in Gini Index was associated with an increased likelihood of both obtaining less than 7 h and 5 h of sleep. Future research should conduct cohort studies, which will allow researchers to determine the temporal relationship between income inequality and sleep over time. This work also points to adverse mental health conditions, such as depression and anxiety, as potential mediators between income inequality and sleep. A better understanding of the mechanisms in which income inequality leads to inadequate sleep. For example, the role of social cohesion and access to mental health services are additional potential mediators that could be investigated. Overall, this study points to the detrimental role of income inequality on mental health, as exhibited through those who reside in states with high income inequality experiencing an increased odds in obtaining inadequate sleep.

## Data Availability

The datasets generated and/or analysed during the current study are available at the BRFSS: https://www.cdc.gov/brfss/annual_data/annual_2018.html.
